# Twenty‐Year Predictors of Peripheral Arterial Disease Compared With Coronary Heart Disease in the Scottish Heart Health Extended Cohort (SHHEC)

**DOI:** 10.1161/JAHA.117.005967

**Published:** 2017-09-18

**Authors:** Hugh Tunstall‐Pedoe, Sanne A. E. Peters, Mark Woodward, Allan D. Struthers, Jill J. F. Belch

**Affiliations:** ^1^ Cardiovascular Epidemiology Unit Institute of Cardiovascular Research University of Dundee United Kingdom; ^2^ Division of Molecular and Clinical Medicine University of Dundee United Kingdom; ^3^ Vascular Medicine Institute of Cardiovascular Research University of Dundee United Kingdom; ^4^ The George Institute for Global Health University of Oxford United Kingdom; ^5^ The George Institute for Global Health University of New South Wales Sydney Australia; ^6^ Department of Epidemiology Johns Hopkins University Baltimore MD

**Keywords:** cohort study, coronary heart disease, epidemiology, peripheral arterial disease, risk prediction, Epidemiology, Risk Factors, Cardiovascular Disease, Primary Prevention

## Abstract

**Background:**

Coronary heart disease and peripheral arterial disease (PAD) affect different vascular territories. Supplementing baseline findings with assays from stored serum, we compared their 20‐year predictors.

**Methods and Results:**

We randomly recruited 15 737 disease‐free men and women aged 30 to 75 years across Scotland between 1984 and 1995 and followed them through 2009 for death and hospital diagnoses. Of these, 3098 developed coronary heart disease (19.7%), and 499 PAD (3.2%). Hazard ratios for 45 variables in the Cox model were adjusted for age and sex and for factors in the 2007 ASSIGN cardiovascular risk score. Forty‐four of them were entered into parsimonious predictive models, tested by c‐statistics and net reclassification improvements. Many hazard ratios diminished with adjustment and parsimonious modeling, leaving significant survivors. The hazard ratios were mostly higher in PAD. New parsimonious models increased the c‐statistic and net reclassification improvements over ASSIGN variables alone but varied in their components and ranking. Coronary heart disease and PAD shared 7 of the 9 factors from ASSIGN: age, sex, family history, socioeconomic status, diabetes mellitus, tobacco smoking, and systolic blood pressure (but neither total nor high‐density lipoprotein cholesterol); plus 4 new ones: NT‐pro‐BNP, cotinine, high‐sensitivity C‐reactive protein, and cystatin‐C. The highest ranked hazard ratios for continuous factors in coronary heart disease were those for age, total cholesterol, high‐sensitivity troponin, NT‐pro‐BNP, cotinine, apolipoprotein A, and waist circumference (plus 10 more); in PAD they were age, high‐sensitivity C‐reactive protein, systolic blood pressure, expired carbon monoxide, cotinine, socioeconomic status, and lipoprotein (a) (plus 5 more).

**Conclusions:**

The mixture of shared with disparate determinants for arterial disease in the heart and the legs implies nonidentical pathogenesis: cholesterol dominant in the former, and inflammation (high‐sensitivity C‐reactive protein, diabetes mellitus, smoking) in the latter.

**Clinical Perspective**

**What Is New?**

- Large prospective studies, with limited risk‐factor measurements, may conclude that risk factors for peripheral arterial disease (PAD) and coronary heart disease are the same.
- The Scottish Heart Health Extended Cohort (SHHEC) study involved measurement of 45 factors with follow‐up of 15 737 men and women over 20 years, generating 3098 cases of coronary heart disease and 499 cases of PAD, enabling detailed comparison of predictors.

**What Are the Clinical Implications?**

- Despite overlap, predictors differed for PAD and coronary heart disease. Total cholesterol, dominant in coronary heart disease, was not predictive in PAD, where inflammatory markers dominated.
- Diabetes mellitus was a powerful predictor of PAD but not the most common cause.
- Tobacco smoking was the predominant cause of PAD, evidenced both by self‐report and several biochemical measures of smoking, which reinforced prediction.
- A 3.2% incidence of clinical cases, over 20 years, does not warrant a separate PAD predictive score in healthy subjects, compared with predicting cardiovascular disease as a whole.
- Differing predictors suggest mechanisms for future interventions.

Occlusive arterial atherothrombosis is common to both coronary heart disease (CHD) and peripheral arterial disease (PAD), but in different vascular territories, usually in different sized arteries. Predictors of the predominant cardiovascular killer diseases CHD and stroke have been extensively investigated, leaving PAD comparatively neglected, although of increasing worldwide importance.^1^, ^2^ Presenting clinically less commonly, and later in life, and commonly missed unless looked for, PAD is harder to study. Despite being associated with serious morbidity, and an ominous marker for diminished survival, even in its much more common presymptomatic form,^3^, ^4^, ^5^ PAD is more likely to be classified as an associated rather than a direct cause of death. Unlike CHD, the literature on risk factors for PAD is dominated, with exceptions, by cross‐sectional and case‐control studies featuring contemporaneous measurements.^1^, ^6^, ^7^ A detailed comparison of predictors of the 2 clinical outcomes, measured in advance of disease, is made possible within the Scottish Heart Health Extended Cohort (SHHEC). Assay of archived serum provides many new biomarkers, and 20 years of follow‐up provide sufficient incident cases to facilitate discrimination of these 2 cardiovascular end points.

## Methods

### Recruitment

SHHEC combines men and women of the Scottish Heart Health Study (SHHS), aged 40 to 59 years, randomly recruited across 23 districts of Scotland in 1984 to 1987,^8^, ^9^ with those of Scottish MONICA aged 25 to 64 years from Edinburgh and North Glasgow 1986; and North Glasgow 1989, 1992 (25 to 75 years), and 1995.^10^ With ethical committee approval, participants gave written consent for the study, completed a self‐administered health record, including the reporting of chest pain^11^ and leg pain on exercise^12^; they attended recruitment survey clinics at which they had physical measurements including brachial but not ankle blood pressure; they each had a 12‐lead resting ECG, categorized using the Minnesota code^13^; gave blood, and consented to follow‐up through their medical records. SHHEC received extra resources for intensive study of smoking and its biochemistry, with a detailed questionnaire, and measurement of expired air carbon monoxide, serum thiocyanate, and serum cotinine.^14^ Each of the 23 SHHS districts was visited twice in different seasons.^8^ North Glasgow MONICA involved 4 independently sampled population surveys at 3‐year intervals.

Baseline cardiovascular disease (CVD) was identified by self‐report and documented hospital discharge diagnoses. It included any diagnosis of CHD; cerebrovascular disease, including transient ischemic attacks; heart failure and PAD, plus major Q waves on the recruitment ECG. Mortality and hospital inpatient episodes were followed by periodic national record linkage. That was used to the end of 2005 in the derivation of the Scottish ASSIGN risk score, a combined cardiovascular end point, used here for comparison with the new prediction models.^15^ Follow‐up to the end of 2009 increased numbers of events, allowing study of CHD and PAD as separate end points.

### Exclusions and End Points

After exclusion of baseline CVD, any CHD or PAD diagnosis during follow‐up in any of the multiple cause fields of hospital diagnoses or of death certification qualified as an end point event; the first recorded event in each category ended follow‐up for that item. CHD was defined as International Classification of Diseases Edition 9 (ICD 9) 410 to 414; (ICD 10) I20 to I25, while PAD was (ICD 9) 440.2, 443.9, 250.6; (ICD 10) I70.2, I73.9, E10.5, E11.5, E12.5, E13.5, E14.5.

### Laboratory Procedures

After venipuncture, blood for serum was left to coagulate up to 4 hours. Separated serum was stored at 4°C pending laboratory testing for recruitment biomarkers 1 to 3 days later in a dedicated Dundee laboratory. Plasma was stored at −40°C and analyzed for fibrinogen in Glasgow^16^ but archived long term in only a minority, whereas aliquots of serum were generally biobanked long term at −40°C and assayed as part of the MORGAM Biomarker study, and BiomarCaRE, in laboratories in Mainz and Hamburg between 2008 and 2014.^17^, ^18^

### Statistical Analyses

Participants were excluded if below age 30 or with existing CVD (see above). Baseline characteristics of the study population were analyzed overall and separately for men and women, without regard to age or missing values. Hazard ratios were then calculated by the Cox proportional hazards method, after proportionality for key risk factors had been confirmed, first adjusted for age and sex, and then multiply adjusted for all 9 variables in the ASSIGN^15^ score. These are age, sex, family history of coronary heart disease, socioeconomic status (from the postcode‐derived deprivation score, the Scottish Index of Multiple Deprivation),^15^ diabetes mellitus, cigarette equivalent dose (incorporating self‐reported cigarette consumption, plus equivalent for pipe and cigar smokers and smoking deceivers using their biochemical results),^14^ systolic blood pressure, total cholesterol and high‐density lipoprotein (HDL) cholesterol. For the hazard‐ratio calculations, multiple imputation was used for missing values, by chained equations with 5 multiple imputation data sets.^19^ Variables were then reduced in number to produce 2 parsimonious models for prediction of each end point. First, the factors were given an equal start whether or not they were ASSIGN risk factors, and stepwise regression was used to produce a “best model,” retaining only those that remained significantly predictive at the 5% level when mutually adjusted. In the second “extended ASSIGN model,” ASSIGN risk factors were forced to remain in while the other factors competed for inclusion. C‐statistics and net reclassification improvements, the latter using 10% and 20% 10‐year risk thresholds, were used to estimate the resulting changes in prediction of the 2 end points with these 2 models, in comparison with the 9 factors previously used in ASSIGN.^20^, ^21^, ^22^ Analyses were conducted using StataSE 12 (Statacorp, College Station, TX), and R version 3.3.0 (R Foundation, Vienna, Austria). Statistical testing was done for main analyses with the sexes combined, but sex‐specific results were also calculated.

## Results

### Numbers Involved

Exclusion from the 18 107 SHHEC participants of those below age 30 (750), and/or those with CVD at recruitment (1623, with 3 overlapping), reduced this to 15 737. Many baseline risk factors were recorded in all of these, but shortage of serum, starting with failed or refused venipuncture, was a limiting factor. Completeness ranged from 90.1% (uric acid) down to 69.8% (cotinine) for initial Dundee assays. For biobanked serum sent to Germany, it was 81.8% (apolipoprotein A) down to 66.8% (NT‐pro‐BNP) as serum became progressively depleted.

### Baseline Status of Participants and Numbers of End Point Events During Follow‐Up

Baseline levels of risk factors derived from the health questionnaire and recruitment survey clinic, from biomarkers measured then, and in archived serum decades later, are shown in Table 1 along with numbers of completed measurements.

**Table 1:** Baseline Variable Summary Statistics: SHHEC Participants by Sex, Aged ≥30, and Free of CVD

	Values Obtained	Men	Women	All
Number of participants	15 737	7552	8185	15 737
Personal				
Age^b^	15 737	49.0 (8.3)	49.0 (8.3)	49.0 (8.3)
Sex,^b^ male%	15 737	100	0	52.0.
Years of education	15 396	11.2 (2.8)	11.1 (2.5)	11.1 (2.6)
Family history of CHD,^b^ %	15 737	25.5	31.8	28.8
SIMD‐socioeconomic score^b^	15 737	28.0 (21.9)	28.6 (22.0)	28.3 (21.9)
ASSIGN cardiovascular risk score^b^	13 444	14.8 (10.6)	9.5 (9.9)	12.1 (10.6)
Smoking				
Current tobacco smoker, %	15 737	51.6	40.2	45.7
Cigarettes equivalent,^b^ cigs/d	15 737	9.1 (11.9)	6.6 (9.6)	7.8 (10.83)
Expired carbon monoxide, ppm	15 257	10.6 (12.5)	9.0 (11.5)	9.8 (12.0)
Thiocyanate, μmol/L^a^	13 738^a^	57.8 (1.99)	58.4 (1.98)	58.1 (1.98)
Cotinine, ng/mL	10 984	128 (171)	103 (152)	115 (162)
Physical measurements				
Height, m	15 729	1.73 (0.07)	1.60 (0.06)	1.66 (0.09)
Weight, kg	15 727	77.9 (12.5)	65.9 (12.6)	71.6 (13.9)
Body mass index, kg/m^2^	15 725	26.0 (3.7)	25.8 (4.8)	25.9 (4.3)
Waist circumference, cm	4061	92.2 (11.6)	80.5 (12.2)	85.9 (13.2)
Waist/hip ratio	4061	0.92 (0.078)	0.79 (0.071)	0.85 (0.096)
SBP,^b^ mm Hg	15 729	134.0 (19.4)	130.5 (21.1)	132.1 (20.4)
DBP, mm Hg	15 729	83.4 (12.0)	79.6 (11.9)	81.4 (12.1)
Pulse pressure, mm Hg	15 729	50.6 (14.1)	50.8 (15.1)	50.7 (14.6)
Pulse rate, bpm	15 661	76.5 (13.3)	78.6 (12.3)	77.6 (12.9)
Diabetes mellitus				
Diabetes mellitus,^b^ %	15 737	1.6	1.4	1.5
Glucose, mmol/L (nonfasting)	12 837	5.17 (1.54)	4.92 (1.36)	5.04 (1.46)
Insulin, μU/mL (nonfasting)^c^	12 281	11.2 (13.2)	8.52 (9.60)	9.83 (11.6)
C‐Peptide, mg/mL^a^, ^c^	12 365 (13)^a^	2.41 (1.92)	2.14 (1.82)	2.27 (1.90)
Lipids				
Total cholesterol,^b^ mmol/L	14 172	6.23 (1.16)	6.40 (1.30)	6.32 (1.24)
HDL‐cholesterol,^b^ mmol/L	13 460	1.35 (0.37)	1.63 (0.43)	1.49 (0.42)
Non‐HDL‐cholesterol, mmol/L	13 459	4.88 (1.14)	4.78 (1.21)	4.88 (1.18)
Triglycerides, mmol/L^a^	14 158	1.91 (1.77)	1.41 (1.69)	1.64 (1.76)
LDL‐cholesterol, mmol/L^c^	10 833	2.65 (1.27)	2.75 (1.19)	2.70 (1.16)
Apolipoprotein A, g/L^c^	12 867	1.49 (0.26)	1.64 (0.30)	1.57 (0.29)
Apolipoprotein B, g/L^c^	12 866	1.18 (0.30)	1.13 (0.32)	1.15 (0.31)
Lipoprotein (a), mg/dL^a^, ^c^	12 773 (88)^a^	9.55 (3.89)	11.81 (3.38)	10.64 (3.64)
Inflammatory				
hsC‐reactive protein, mg/L^a^, ^c^	12 847	1.53 (2.99)	1.27 (3.10)	1.39 (3.06)
Fibrinogen, g/L	13 101	2.75 (0.80)	2.86 (0.81)	2.81 (0.81)
Homocysteine, μmol/L^c^	12 843	16.0 (6.6)	14.0 (5.7)	14.9 (6.2)
Ferritin, μmol/L^a^, ^c^	12 319 (22)^a^	103.0 (2.64)	38.0 (2.97)	62.0 (3.14)
Cardiac				
hsTroponin I, pg/mL^a^, ^c^	11 527 (427)^a^	3.76 (2.60)	2.25 (3.80)	2.97 (3.29)
NT‐pro‐BNP, pg/mL^a^, ^c^	10 507	31.7 (2.94)	61.5 (2.45)	44.7 (2.83)
Diet related				
25‐Hydroxyvitamin D raw, nmol/L^c^	11 603	44.3 (22.7)	39.2 (19.7)	41.6 (21.3)
25‐Hydroxyvitamin D adj, nmol/L^c^	11 603	44.0 (19.3)	39.4 (17.9)	41.6 (18.7)
Vitamin B_12_, pg/mL^c^	12 355	425 (180)	423 (191)	424 (186)
γ‐Glutamyl transferase, units/L^a^	12 835	31.4 (1.92)	18.5 (1.79)	24.1 (1.96)
Renal related				
Uric acid, mmol/L	14 178	320 (66)	243 (62)	281 (75)
Creatinine, mmol/L	11 837	95.9 (12.9)	81.4 (11.0)	88.6 (14.0)
Cystatin‐C, mg/L^c^	12 868	0.760 (0.143)	0.716 (0.165)	0.738 (0.156)

Missing values are omitted. Percentages are shown for categorical factors. Arithmetic means and standard deviations are shown for continuous variables with some exceptions.^a^CHD indicates coronary heart disease; CVD, cardiovascular disease; DBP, diastolic blood pressure; HDL, high‐density lipoprotein; SBP, systolic blood pressure; SHHEC, Scottish Heart Health Extended Cohort; SIMD, Scottish Index of Multiple Deprivation.

a Geometric means and standard deviations because of positive skew. For these geometric calculations any 0 values (number in parentheses) are set to 0.01 units.

b ASSIGN risk score factor.

c Assayed in the MORGAM/BiomarCaRE project after long‐term storage of serum at −40°C.

The cohort was followed to the end of 2009 from a decade (1984 to 1995) during which Scotland at times had the world's highest coronary heart disease mortality in both sexes. Mean follow‐up of the 15 737 study participants was 19.9 years; of the 11 706 who remained alive at December 31, 2009, it was 22.0 years. Participants from the original 15 737 later recorded as developing any death certificate or hospital inpatient diagnosis of CHD numbered 3098 (19.7%), and of PAD 499 (3.2%). Of the latter, 285 (57%) developed both pathologies—CHD first in 143, PAD first in 97, and both together in 45.

### Prediction From Symptom Questionnaires and the ECG

At recruitment, of the 15 737 without diagnosed CVD, 860 (5.5%) were chest pain positive to the Rose “angina” questionnaire,^11^ of whom 279 later developed clinical CHD and 37 developed PAD; 335 (2.1%) were positive for the Edinburgh leg pain “claudication” questionnaire,^12^ of whom 104 developed CHD and 47 developed PAD (Table 2). Symptom questionnaires anticipated 1 in 11 related events over 20 years. Chest pain questionnaire positive responses had a 1.7‐fold increased CHD incidence over negatives; leg pain positives 4.8‐fold PAD incidence over negatives but developed numerically more cases of CHD than PAD.

**Table 2:** Sensitivity, Specificity, and Predictive Value for Baseline Symptom Questionnaires and ECG Ischemia Versus 20‐Year Disease Outcomes, Sexes Combined

	See Key	Rose Angina Questionnaire		Edinburgh Claudication Questionnaire		Electrocardiographic “Possible Ischemia” (Minnesota Code)	
End Point		CHD	PAD	CHD	PAD	CHD	PAD
True positive N	a	279	37	104	47	382	58
False positive N	b	581	823	231	288	783	1107
False negative N	c	2819	462	2994	452	2716	441
True negative N	d	12 058	14 415	12 408	14 950	11 856	14 131
Sensitivity, %	a×100/(a+c)	9.0	7.4	3.4	9.4	12.3	11.6
Specificity, %	d×100/(b+d)	95.4	94.6	98.2	98.1	93.8	92.7
Positive predictive value, %	a×100/(a+b)	32.4	4.3	31.0	14.0	32.8	5.0
Negative predictive value, %	d×100/(c+d)	81.1	96.9	80.6	97.1	81.4	97.0
Prediction ratio	ppv/(100−npv)	1.7	1.4	1.6	4.8	1.8	1.6

CHD indicates coronary heart disease; PAD, peripheral arterial disease.

**Table:** 

		Disease		
Key		Positive	Negative	
Test	Positive	a	b	a+b
	Negative	c	d	c+d
		a+c	b+d	All 15 737

The 12‐lead ECG, coded to the Minnesota code, was classified in 1165 (7.4%) as “possible ischemia” for minor Q waves and ST and T wave findings.^13^ These findings anticipated 382 clinical CHD, and 58 PAD cases—one in 8 of CHD and of PAD, marginally more than the questionnaires. Positives had a 1.8‐fold increased incidence of CHD over negatives and a 1.6‐fold increase for PAD (Table 2).

### Hazard Ratios

Table 3 shows the Cox proportional hazards ratios (HRs) for CHD and for PAD for the 45 individual, derived, or composite factors from Table 1, initially adjusted by age and sex, for the sexes combined, with missing values imputed. Then this is repeated with additional adjustment for the remaining ASSIGN factors, after omitting these factors and their derivatives, leaving 33 factors. To help comparison, reciprocals of hazard ratios are used in the few variables where that for CHD is <1.0 (for example HDL‐cholesterol). Sex‐specific results are given in Table 4.

**Table 3:** Hazard Ratios and 95% Confidence Limits by Individual Factors for CHD and PAD (Sexes Combined) Adjusted for Age and Sex and Additionally for ASSIGN Factors (Which are Excluded)

		Adjusted for Age and Sex				Adjusted Also for ASSIGN Variables (Which are Excluded)		
		CHD	PAD	PAD/CHD		CHD	PAD	PAD/CHD
Number		3098	499	3098/499		3098	499	3098/499
Personal								
Age^b^		1.68 (1.61; 1.74)^a^	1.84 (1.67; 2.02)^a^	1.10 (1.01; 1.19)^a^		…	…	…
Sex (male)^b^		1.82 (1.69; 1.96)^a^	1.67 (1.41; 2.00)^a^	0.93 (0.80; 1.11)		…	…	…
Years of education	R	1.22 (1.16; 1.27)^a^	1.30 (1.16; 1.45)^a^	1.06 (0.96; 1.19)	R	1.11 (1.06; 1.16)^a^	1.09 (0.97; 1.22)	0.97 (0.88; 1.10)
Family history of CHD^b^		1.55 (1.44; 1.66)^a^	1.53 (1.27; 1.84)^a^	0.99 (0.80; 1.18)		…	…	…
SIMD (deprivation) score^b^		1.23 (1.19; 1.27)^a^	1.51 (1.40; 1.64)^a^	1.23 (1.16; 1.31)^a^		…	…	…
ASSIGN (cardiovascular risk) score^b^		1.47 (1.42; 1.52)^a^	1.78 (1.66; 1.90)^a^	1.21 (1.17; 1.25)^a^		…	…	…
Smoking								
Current tobacco smoker		1.70 (1.58; 1.83)^a^	5.12 (4.14; 6.33)^a^	3.01 (2.80; 3.22)^a^		…	…	…
Cigarettes/d equivalent^b^		1.32 (1.28; 1.37)^a^	1.81 (1.70; 1.93)^a^	1.37 (1.31; 1.43)^a^		…	…	…
Expired air carbon monoxide		1.27 (1.23; 1.31)^a^	1.71 (1.62; 1.81)^a^	1.35 (1.29; 1.41)^a^		1.08 (1.03; 1.13)^a^	1.42 (1.30; 1.54)^a^	1.31 (1.22; 1.40)^a^
Thiocyanate		1.29 (1.24; 1.34)^a^	2.21 (2.00; 2.43)^a^	1.71 (1.61; 1.81)^a^		1.06 (1.00; 1.12)^a^	1.76 (1.53; 2.03)^a^	1.66 (1.51; 1.81)^a^
Cotinine		1.34 (1.30; 1.38)^a^	1.89 (1.75; 2.04)^a^	1.41 (1.34; 1.47)^a^		1.19 (1.12; 1.26)^a^	1.54 (1.35; 1.76)^a^	1.30 (1.19; 1.40)^a^
Physical measurements								
Height	R	1.19 (1.14; 1.25)	1.19 (1.05; 1.35)^a^	1.00 (0.89; 1.14)	R	1.10 (1.05; 1.16)^a^	1.02 (0.90; 1.16)	0.93 (0.82; 1.05)
Weight		1.17 (1.13; 1.22)^a^	1.02 (0.92; 1.13)	0.87 (0.74; 1.00)^a^		1.10 (1.06; 1.15)^a^	0.96 (0.87; 1.06)	0.87 (0.75; 0.99)^a^
Body mass index		1.24 (1.20; 1.28)^a^	1.09 (0.99; 1.19)	0.88 (0.76; 0.99)^a^		1.14 (1.10; 1.18)^a^	0.97 (0.88; 1.06)	0.85 (0.73; 0.96)^a^
Waist circumference		1.31 (1.24; 1.38)^a^	1.22 (1.09; 1.36)^a^	0.93 (0.82; 1.04)		1.18 (1.11; 1.26)^a^	1.03 (0.91; 1.17)	0.87 (0.75; 0.99)^a^
Waist‐to‐hip ratio		1.19 (1.14; 1.25)^a^	1.25 (1.07; 1.45)^a^	1.05 (0.98; 1.11)		1.09 (1.02; 1.16)^a^	1.09 (0.91; 1.30)	1.00 (0.90; 1.10)
Systolic blood pressure^b^		1.24 (1.20; 1.28)^a^	1.46 (1.35; 1.58)^a^	1.17 (1.09; 1.25)^a^		…	…	…
Diastolic blood pressure		1.19 (1.15; 1.24)^a^	1.16 (1.06; 1.26)^a^	0.97 (0.88; 1.06)		1.05 (1.00; 1.10)^a^	0.90 (0.82; 0.99)^a^	0.86 (0.76; 0.95)^a^
Pulse pressure		1.05 (1.03; 1.07)^a^	1.08 (1.05; 1.12)^a^	1.03 (1.02; 1.04)^a^	R	1.10 (1.01; 1.19)	1.02 (0.95; 1.09)	0.93 (0.85; 1.01)
Pulse rate		1.09 (1.05; 1.13)^a^	1.23 (1.14; 1.34)^a^	1.13 (1.05; 1.22)^a^		1.00 (0.96; 1.03)	1.06 (0.97; 1.15)	1.06 (0.98; 1.15)
Diabetes mellitus								
Diabetes mellitus, Y/N^b^		2.63 (2.16; 3.21)	6.76 (4.88; 9.35)	2.57 (2.22; 2.91)		…	…	…
Glucose (nonfasting)		1.11 (1.08; 1.14)	1.23 (1.18; 1.29)	1.11 (1.07; 1.15)		1.05 (1.01; 1.08)	1.17 (1.10; 1.24)	1.12 (1.06; 1.17)
Insulin (nonfasting)		1.09 (1.07; 1.13)	1.13 (1.06; 1.20)	1.03 (0.97; 1.09)		1.06 (1.03; 1.09)	1.07 (0.98; 1.15)	1.00 (0.93; 1.08)
C‐peptide		1.03 (0.97; 1.09)	0.98 (0.88; 1.10)	0.96 (0.79; 1.12)		1.02 (0.97; 1.06)	1.03 (0.95; 1.11)	1.01 (0.88; 1.14)
Lipids								
Total cholesterol^b^		1.27 (1.22; 1.31)^a^	1.18 (1.07; 1.29)^a^	0.93 (0.84; 1.02)		…	…	…
HDL‐cholesterol^b^	R	1.16 (1.12; 1.22)^a^	1.18 (1.05; 1.30)^a^	1.00 (0.91; 1.11)		…	…	…
Non‐HDL‐cholesterol		1.34 (1.29; 1.38)^a^	1.24 (1.14; 1.36)^a^	0.93 (0.84; 1.02)		…	…	…
LDL‐cholesterol		1.14 (1.10; 1.18)^a^	1.10 (1.01; 1.21)^a^	0.97 (0.89; 1.04)	R	1.01 (0.96; 1.05)	1.02 (0.91; 1.14)	1.01 (0.92; 1.12)
Triglycerides		1.35 (1.30; 1.41)^a^	1.47 (1.33; 1.63)^a^	1.09 (1.00; 1.18)^a^		1.13 (1.08; 1.18)^a^	1.19 (1.07; 1.33)^a^	1.05 (0.95; 1.16)
Apolipoprotein A	R	1.22 (1.16; 1.27)^a^	1.23 (1.09; 1.41)^a^	1.02 (0.93; 1.14)	R	1.14 (1.08; 1.20)^a^	1.12 (0.96; 1.30)	0.98 (0.87; 1.12)
Apolipoprotein B		1.34 (1.29; 1.38)^a^	1.32 (1.23; 1.43)^a^	0.99 (0.92; 1.06)		1.15 (1.08; 1.23)^a^	1.15 (0.99; 1.33)	1.00 (0.86; 1.13)
Lipoprotein (a)		1.03 (0.99; 1.07)	1.22 (1.03; 1.44)^a^	1.19 (0.95; 1.42)		1.01 (0.97; 1.05)	1.19 (1.02; 1.40)^a^	1.18 (0.96; 1.40)
Inflammatory								
hsC‐reactive protein		1.38 (1.31; 1.45)^a^	1.82 (1.66; 1.99)^a^	1.32 (1.24; 1.40)^a^		1.22 (1.15; 1.29)^a^	1.48 (1.33; 1.64)^a^	1.21 (1.11; 1.32)^a^
Fibrinogen		1.18 (1.14; 1.22)^a^	1.31 (1.23; 1.39)^a^	1.11 (1.05; 1.17)^a^		1.09 (1.05; 1.13)^a^	1.17 (1.09; 1.27)^a^	1.08 (1.00; 1.15)^a^
Homocysteine		1.15 (1.11; 1.19)^a^	1.21 (1.12; 1.30)^a^	1.05 (0.99; 1.11)		1.08 (1.05; 1.12)^a^	1.07 (0.98; 1.15)	0.98 (0.91; 1.06)
Ferritin		1.07 (1.01; 1.13)^a^	1.23 (0.95; 1.59)	1.15 (0.98; 1.32)		1.02 (0.97; 1.07)	1.07 (0.88; 1.31)	1.05 (0.92; 1.18)
Cardiac								
hsTroponin I		1.60 (1.43; 1.77)^a^	1.78 (1.26; 2.29)^a^	1.11 (0.44; 1.78)		1.29 (1.14; 1.53)^a^	1.30 (0.97; 1.65)	1.01 (0.59; 1.44)
NT‐pro‐BNP		1.24 (1.18; 1.30)^a^	1.40 (1.24; 1.58)^a^	1.12 (1.02; 1.23)^a^		1.21 (1.16; 1.27)^a^	1.25 (1.11; 1.40)^a^	1.03 (0.92; 1.13)
Diet related								
25OHD raw	R	1.18 (1.11; 1.25)^a^	1.61 (1.39; 1.89)^a^	1.37 (1.19; 1.61)^a^	R	1.05 (0.99; 1.11)	1.28 (1.10; 1.49)^a^	1.22 (1.06; 1.41)^a^
25OHD adjusted	R	1.18 (1.11; 1.25)^a^	1.59 (1.39; 1.85)^a^	1.37 (1.18; 1.61)^a^	R	1.05 (0.99; 1.11)	1.20 (1.08; 1.45)^a^	1.20 (1.05; 1.39)^a^
Vitamin B_12_		1.02 (0.97; 1.06)	1.05 (0.96; 1.16)	1.04 (0.95; 1.13)		1.01 (0.97; 1.06)	0.98 (0.89; 1.08)	0.96 (0.89; 1.05)
γ‐Glutamyl transferase		1.20 (1.15; 1.25)^a^	1.41 (1.29; 1.53)^a^	1.17 (1.09; 1.13)^a^		1.07 (1.02; 1.13)^a^	1.19 (1.08; 1.31)^a^	1.11 (1.02; 1.20)^a^
Renal related								
Uric acid		1.09 (1.04; 1.13)^a^	1.02 (0.91; 1.15)	0.94 (0.82; 1.06)		1.04 (1.00; 1.09)^a^	1.03 (0.91; 1.17)	0.99 (0.87; 1.11)
Creatinine		1.01 (0.96; 1.07)	1.00 (0.87; 1.14)	0.99 (0.84; 1.14)		1.03 (0.98; 1.08)	1.08 (0.97; 1.20)	1.05 (0.94; 1.16)
Cystatin‐C		1.16 (1.13; 1.18)^a^	1.19 (1.15; 1.23)^a^	1.03 (0.96; 1.10)		1.13 (1.10; 1.16)^a^	1.18 (1.11; 1.25)^a^	1.05 (0.98; 1.11)

Missing values are imputed using multiple imputation by chained equations (m=5, i=10). Hazard ratios are per standard deviation for continuous variables; some are log transformed (see Table 1). CHD indicates coronary heart disease; HDL, high‐density lipoprotein; hs, high sensitivity; LDL, low‐density lipoprotein; PAD, peripheral arterial disease; R, hazard ratios converted to their reciprocals to facilitate comparison (using CHD as the criterion); SIMD, Scottish Index of Multiple Deprivation; 25OHD, 25‐hydroxyvitamin D.

a Hazard ratios significant at 5%.

b ASSIGN risk score factors.

**Table 4:** Hazard Ratios and 95% Confidence Limits by Individual Factors for CHD and PAD, Adjusted First for Age and Then Additionally for ASSIGN Factors (Which are Excluded), for the Sexes Separately

Adjusted for Age		Coronary Heart Disease			Peripheral Arterial Disease	
		Men 1847	Women 1251		Men 291	Women 208
Personal						
Age^b^		1.57 (1.50; 1.65)^a^	1.84 (1.73; 1.95)^a^		1.75 (1.54; 1.99)^a^	1.96 (1.69; 2.28)^a^
Years of education	R	1.16 (1.11; 1.22)^a^	1.32 (1.23; 1.43)^a^	R	1.27 (1.11; 1.45)^a^	1.35 (1.12; 1.61)^a^
Family history CHD^b^		1.45 (1.31; 1.60)^a^	1.69 (1.51; 1.89)^a^		1.57 (1.23; 2.01)^a^	1.48 (1.12; 1.95)^a^
SIMD score (SES)^b^		1.18 (1.12; 1.23)^a^	1.30 (1.23; 1.37)^a^		1.48 (1.33; 1.65)^a^	1.56 (1.38; 1.77)^a^
ASSIGN CV risk score^b^		1.44 (1.39; 1.50)^a^	1.50 (1.44; 1.56)^a^		1.76 (1.62; 1.92)^a^	1.79 (1.66; 1.93)^a^
Smoking						
Current smoker Y/N		1.60 (1.46; 1.76)^a^	1.86 (1.66; 2.07)^a^		3.96 (3.01; 5.21)^a^	7.08 (5.07; 9.88)^a^
Cigs/day equivalent^b^		1.29 (1.24; 1.34)^a^	1.40 (1.33; 1.48)^a^		1.68 (1.54; 1.82)^a^	2.01 (1.85; 2.19)^a^
Expired CO		1.26 (1.22; 1.31)^a^	1.28 (1.21; 1.34)^a^		1.61 (1.49; 1.73)^a^	1.88 (1.73; 2.04)^a^
Thiocyanate		1.28 (1.22; 1.34)^a^	1.30 (1.22; 1.37)^a^		2.05 (1.81; 2.33)^a^	2.44 (2.09; 2.85)^a^
Cotinine		1.33 (1.27; 1.38)^a^	1.37 (1.30; 1.44)^a^		1.76 (1.59; 1.95)^a^	2.09 (1.87; 2.34)^a^
Physical						
Height	R	1.15 (1.08; 1.22)^a^	1.28 (1.19; 1.41)^a^	R	1.18 (1.01; 1.37)^a^	1.20 (0.98; 1.49)
Weight		1.14 (1.08; 1.20)^a^	1.21 (1.14; 1.28)^a^		0.93 (0.81; 1.07)	1.15 (0.99; 1.33)
Body mass index		1.24 (1.17; 1.30)^a^	1.24 (1.18; 1.29)^a^		0.99 (0.86; 1.14)	1.17 (1.04; 1.31)^a^
Waist circumference.		1.27 (1.19; 1.36)^a^	1.36 (1.27; 1.45)^a^		1.09 (0.93; 1.29)	1.37 (1.16; 1.61)^a^
Waist‐to‐hip ratio		1.16 (1.09; 1.22)^a^	1.27 (1.18; 1.37)^a^		1.14 (0.98; 1.33)	1.42 (1.17; 1.71)^a^
Systolic BP^b^		1.23 (1.18; 1.29)^a^	1.26 (1.19; 1.32)^a^		1.47 (1.33; 1.63)^a^	1.44 (1.28; 1.61)^a^
Diastolic BP		1.19 (1.14; 1.25)^a^	1.19 (1.13; 1.26)^a^		1.18 (1.06; 1.32)^a^	1.12 (0.97; 1.29)
Pulse pressure		1.05 (1.02; 1.08)^a^	1.05 (1.02; 1.08)^a^		1.09 (1.05; 1.14)^a^	1.08 (1.03; 1.12)^a^
Pulse rate		1.09 (1.05; 1.13)^a^	1.09 (1.05; 1.13)^a^		1.23 (1.14; 1.34)^a^	1.23 (1.14; 1.34)^a^
Diabetes						
Diabetes mellitus^b^		2.26 (1.72; 2.96)^a^	3.24 (2.42; 4.32)^a^		6.31 (4.08; 9.76)^a^	7.39 (4.55; 12.00)^a^
Glucose (non‐fasting)		1.09 (1.04; 1.13)^a^	1.15 (1.09; 1.21)^a^		1.24 (1.17; 1.32)^a^	1.23 (1.13; 1.34)^a^
Insulin (non‐fasting)		1.07 (1.03; 1.10)^a^	1.15 (1.11; 1.19)^a^		1.06 (0.96; 1.16)	1.20 (1.12; 1.29)^a^
C‐peptide		1.05 (0.98; 1.13)^a^	1.01 (0.91; 1.12)		1.00 (0.86; 1.17)	0.98 (0.81; 1.18)
Lipids						
Total cholesterol^b^		1.30 (1.24; 1.36)^a^	1.23 (1.16; 1.29)^a^		1.13 (0.99; 1.28)	1.23 (1.07; 1.41)^a^
HDL cholesterol^b^	R	1.15 (1.09; 1.20)^a^	1.20 (1.14; 1.27)^a^	R	1.02 (0.91; 1.15)	1.37 (1.15; 1.64)^a^
Non‐HDL cholesterol		1.36 (1.30; 1.43)^a^	1.31 (1.24; 1.38)^a^		1.14 (1.00; 1.28)^a^	1.37 (1.20; 1.56)^a^
LDL cholesterol		1.13 (1.08; 1.18)^a^	1.15 (1.09; 1.22)^a^		1.10 (0.98; 1.24)	1.11 (0.98; 1.26)
Triglycerides		1.30 (1.23; 1.36)^a^	1.45 (1.36; 1.55)^a^		1.26 (1.12; 1.42)^a^	1.85 (1.58; 2.18)^a^
Apolipoprotein A	R	1.18 (1.12; 1.25)^a^	1.27 (1.19; 1.35)^a^	R	1.20 (1.04; 1.39)^a^	1.28 (1.05; 1.54)^a^
Apolipoprotein B		1.38 (1.31; 1.46)^a^	1.29 (1.24; 1.35)^a^		1.28 (1.15; 1.44)^a^	1.35 (1.23; 1.50)^a^
Lipoprotein (a)		1.01 (0.97; 1.06)	1.10 (0.98; 1.24)		1.12 (0.94; 1.34)	1.59 (0.97; 2.61)
Inflammatory						
hsC‐reactive protein		1.32 (1.24; 1.40)^a^	1.47 (1.37; 1.58)^a^		1.73 (1.53; 1.95)^a^	1.95 (1.70; 2.24)^a^
Fibrinogen		1.17 (1.12; 1.22)^a^	1.20 (1.13; 1.27)^a^		1.32 (1.23; 1.42)^a^	1.29 (1.15; 1.44)^a^
Homocysteine		1.16 (1.11; 1.21)^a^	1.14 (1.06; 1.22)^a^		1.20 (1.09; 1.32)^a^	1.22 (1.08; 1.39)^a^
Ferritin		1.04 (0.98; 1.11)	1.12 (1.01; 1.23)^a^		1.21 (0.97; 1.50)	1.26 (0.83; 1.91)
Cardiac						
hsTroponin I		1.68 (1.39; 1.98)^a^	1.57 (1.41; 1.73)^a^		2.13 (1.12; 3.14)^a^	1.64 (1.04; 2.24)^a^
NT‐pro‐BNP		1.23 (1.17; 1.30)^a^	1.27 (1.17; 1.38)^a^		1.44 (1.27; 1.63)^a^	1.32 (1.07; 1.64)^a^
Diet related						
25OH‐vitamin D raw	R	1.19 (1.10; 1.28)^a^	1.16 (1.09; 1.25)^a^	R	1.67 (1.43; 1.96)^a^	1.52 (1.14; 2.00)^a^
25OH‐vitamin D adj	R	1.16 (1.09; 1.25)^a^	1.20 (1.11; 1.30)^a^	R	1.61 (1.37; 1.89)^a^	1.59 (1.16; 2.17)^a^
Vitamin B12		1.03 (0.97; 1.09)	1.00 (0.94; 1.06)		1.07 (0.95; 1.21)	1.03 (0.90; 1.18)
Gamma glutamyl TF		1.17 (1.11; 1.23)^a^	1.27 (1.19; 1.35)^a^		1.32 (1.18; 1.48)^a^	1.54 (1.36; 1.76)^a^
Renal related						
Uric Acid		1.02 (0.97; 1.09)	1.19 (1.11; 1.27)^a^		0.91 (0.78; 1.06)	1.21 (1.03; 1.42)^a^
Creatinine		0.99 (0.93; 1.06)	1.05 (0.97; 1.12)		0.99 (0.85; 1.15)	1.02 (0.82; 1.26)
Cystatin ‐C		1.20 (1.14; 1.26)^a^	1.14 (1.12; 1.17)^a^		1.26 (1.15; 1.40)^a^	1.17 (1.12; 1.23)^a^

Adjusted Additionally for ASSIGN Variables (Which are Omitted)						
		Coronary Heart Disease			Peripheral Artery Disease	
		Men 1847	Women 1251		Men 291	Women 208
Personal						
Years of education	R	1.10 (1.04; 1.16)^a^	1.15 (1.08; 1.23)^a^	R	1.11 (0.96; 1.28)	1.04 (0.87; 1.25)
Smoking						
Expired CO		1.09 (1.03; 1.14)^a^	1.08 (1.01; 1.15)^a^		1.33 (1.20; 1.47)^a^	1.56 (1.40; 1.73)^a^
Thiocyanate		1.05 (0.99; 1.12)	1.07 (1.00; 1.15)^a^		1.63 (1.38; 1.92)^a^	1.94 (1.62; 2.32)^a^
Cotinine		1.18 (1.11; 1.27)^a^	1.19 (1.12; 1.28)^a^		1.42 (1.22; 1.66)^a^	1.72 (1.48; 1.99)^a^
Physical						
Height	R	1.08 (1.01; 1.14)^a^	1.16 (1.08; 1.27)^a^	R	1.03 (0.89; 1.22)	1.00 (0.81; 1.23)
Weight		1.09 (1.04; 1.15)^a^	1.12 (1.05; 1.19)^a^		0.91 (0.80; 1.04)	1.03 (0.89; 1.19)
Body mass index		1.14 (1.09; 1.21)^a^	1.14 (1.09; 1.20)^a^		0.90 (0.79; 1.04)	1.02 (0.91; 1.15)
Waist circumference		1.17 (1.08; 1.26)^a^	1.20 (1.11; 1.30)^a^		0.97 (0.82; 1.15)	1.11 (0.93; 1.33)
Waist‐to‐hip ratio		1.07 (0.99; 1.16)	1.12 (1.02; 1.23)^a^		1.03 (0.87; 1.23)	1.17 (0.91; 1.51)
Diastolic BP		1.06 (1.00; 1.12)^a^	1.03 (0.97; 1.10)		0.91 (0.81; 1.03)	0.88 (0.78; 1.00)^a^
Pulse pressure	R	1.11 (1.00; 1.23)^a^	1.09 (0.99; 1.20)	R	0.99 (0.91; 1.09)	1.03 (0.94; 1.12)
Pulse rate		1.00 (0.96; 1.03)	1.00 (0.96; 1.03)		1.06 (0.97; 1.15)	1.06 (0.97; 1.15)
Diabetes						
Glucose (non‐fasting)		1.03 (0.99; 1.07)	1.08 (1.03; 1.13)^a^		1.19 (1.10; 1.28)^a^	1.14 (1.04; 1.24)^a^
Insulin (non‐fasting)		1.05 (1.01; 1.08)^a^	1.09 (1.04; 1.14)^a^		1.04 (0.93; 1.16)	1.09 (0.98; 1.20)
C‐peptide		1.03 (0.98; 1.10)	1.01 (0.94; 1.08)		1.05 (0.93; 1.20)	1.01 (0.90; 1.13)
Lipids						
LDL cholesterol	R	1.00 (0.94; 1.05)	1.02 (0.95; 1.09)	R	1.00 (0.88; 1.15)	1.04 (0.90; 1.20)
Triglycerides		1.12 (1.06; 1.18)^a^	1.15 (1.07; 1.24)^a^		1.08 (0.95; 1.22)	1.41 (1.19; 1.67)^a^
Apolipoprotein A		1.11 (1.04; 1.18)^a^	1.18 (1.08; 1.27)^a^		1.11 (0.93; 1.32)	1.12 (0.92; 1.37)
Apolipoprotein B	R	1.21 (1.11; 1.31)^a^	1.10 (1.02; 1.18)^a^	R	1.14 (0.96; 1.36)	1.15 (0.98; 1.36)
Lipoprotein (a)		1.01 (0.97; 1.05)	1.02 (0.93; 1.12)		1.13 (0.95; 1.33)	1.47 (0.89; 2.41)
Inflammatory						
hsC‐reactive protein		1.17 (1.08; 1.26)^a^	1.28 (1.19; 1.38)^a^		1.41 (1.23; 1.62)^a^	1.57 (1.35; 1.82)^a^
Fibrinogen		1.09 (1.04; 1.14)^a^	1.09 (1.02; 1.16)^a^		1.21 (1.10; 1.34)^a^	1.12 (0.99; 1.27)
Homocysteine		1.10 (1.05; 1.14)^a^	1.05 (0.98; 1.13)		1.06 (0.96; 1.17)	1.08 (0.94; 1.25)
Ferritin		1.01 (0.95; 1.07)	1.03 (0.95; 1.12)		1.08 (0.90; 1.31)	1.07 (0.80; 1.43)
Cardiac						
hsTroponin I		1.33 (1.14; 1.41)^a^	1.28 (1.16; 1.40)^a^		1.51 (0.79; 2.23)	1.24 (0.85; 1.63)
NT‐pro‐BNP		1.21 (1.15; 1.27)^a^	1.23 (1.13; 1.34)^a^		1.27 (1.13; 1.44)^a^	1.20 (0.97; 1.49)
Diet related						
25OH‐vitamin D raw	R	1.05 (0.99; 1.12)	1.04 (0.96; 1.11)	R	1.35 (1.12; 1.61)^a^	1.16 (0.89; 1.54)
25OH‐vitamin D adj.	R	1.06 (1.00; 1.14)^a^	1.02 (0.94; 1.09)	R	1.28 (1.09; 1.52)^a^	1.20 (0.89; 1.61)
Vitamin B12	R	1.00 (0.93; 1.05)^a^	1.03 (0.97; 1.10)	R	0.97 (0.85; 1.10)	0.99 (0.86; 1.14)
Gamma glutamyl TF		1.05 (1.00; 1.11)^a^	1.11 (1.03; 1.20)^a^		1.13 (1.00; 1.28)^a^	1.30 (1.13; 1.49)^a^
Renal related						
Uric Acid		1.02 (0.96; 1.08)	1.08 (1.01; 1.16)^a^		0.96 (0.82; 1.13)	1.14 (0.96; 1.34)
Creatinine		1.03 (0.97; 1.10)	1.03 (0.96; 1.10)		1.09 (0.97; 1.22)	1.06 (0.87; 1.30)
Cystatin‐C		1.14 (1.09; 1.20)^a^	1.12 (1.07; 1.16)^a^		1.18 (1.07; 1.30)^a^	1.18 (1.10; 1.27)^a^

These are sex‐specific results equivalent to the sexes combined results in Table 3. Missing values are imputed using multiple imputation by chained equation (m=5, i=10). Hazard ratios are per standard deviation for continuous variables, some are log transformed (see Table 1). R‐Hazard ratios <1.0 are converted to their reciprocal to facilitate comparison (using CHD both sexes as the criterion from Table 3).

a Hazard ratios significant at 5%.

b ASSIGN risk score factors.

In Table 3 almost all HRs are initially significant at 5%: 41 out of 45 for CHD, and 38 out of 45 for PAD despite smaller numbers and consequently bigger confidence intervals.

Comparison of the hazard ratios in Table 3 between PAD and CHD, shows PAD HRs initially higher in 30, significantly so in 22. This applies to 5 of the 9 ASSIGN factors, specifically to age, socioeconomic status, smoking (but also all measures of smoking biochemistry), systolic blood pressure (but also pulse pressure and pulse rate), diabetes mellitus, and also nonfasting glucose, triglycerides, high‐sensitivity C‐reactive protein (hsCRP), fibrinogen, NT‐pro‐BNP, 25‐hydroxyvitamin D (25OHD), and γ‐glutamyl transferase. However, among the 8 lipid variables, only the HR for triglycerides is significantly different in this analysis, although that for lipoprotein (a) emerges in later analyses. In only 13 variables were HRs for CHD more extreme than those for PAD, 2 significantly so: weight and body mass index (BMI).

After adjustment for ASSIGN variables in Table 3, there are 24 remaining significant factors for CHD out of 33, and the number for PAD is 14. The HRs remain significantly increased in PAD compared with CHD for the 3 measures of smoke inhalation, nonfasting glucose, hsCRP, fibrinogen, 25OHD, and γ‐glutamyl transferase. None of the 5 remaining lipids shows a significant difference. Adjustment for ASSIGN variables has reversed the HR gradient for pulse pressure, LDL‐cholesterol, and B_12_, now shown as the reciprocal, but not significantly different between the end points. There are 10 factors for which CHD HRs are more extreme, weight and BMI significantly so, with waist circumference now added, and diastolic blood pressure anomalous—the HRs for the 2 diseases are both significant, but significantly different, on either side of 1.00 (see Discussion).

### Parsimonious Modeling

Stepwise elimination of factors for parsimonious models was not contingent on analyses for Table 3, as almost every factor was entered anew into these calculations (the exception was non‐HDL‐cholesterol, removed because it was derived by subtraction, causing unacceptable collinearity with total cholesterol). In the Figure factors are displayed for the best models for CHD and PAD in descending order of hazard ratios for both sexes combined, using the reciprocal for those <1.0 and separating binary and continuous variables. CHD has 20 factors in the best model, and PAD 16 despite many fewer end points. Again PAD has higher HRs than CHD, with wider confidence intervals. However, the adjustment of each HR for all others in the model means that individual HRs should not now be compared directly. Data for this figure, for sex‐specific results, and also for the extended ASSIGN models are available in Table 5.

**Figure 1:**
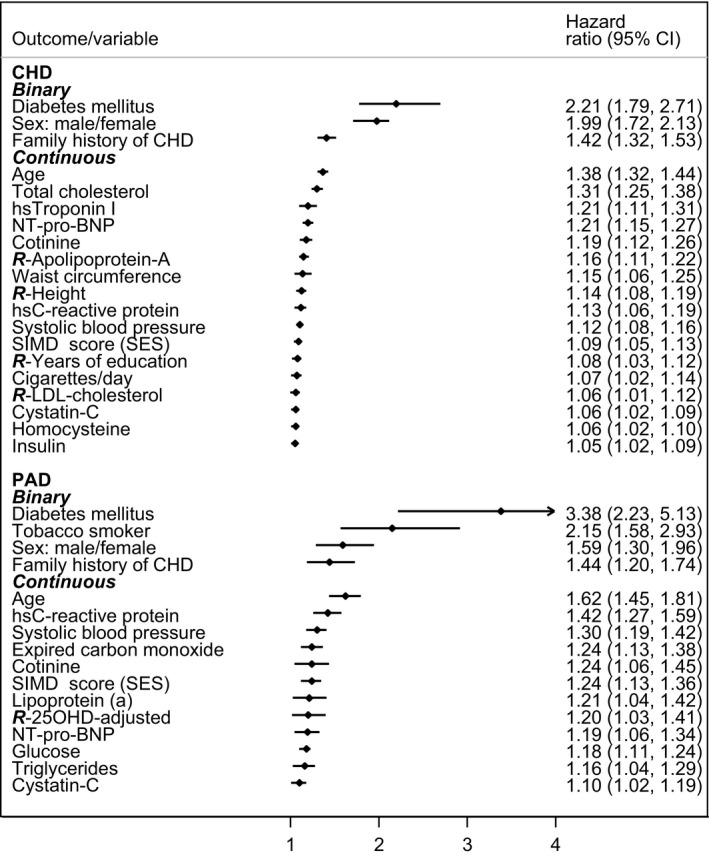
Ranking of hazard ratios (shown with 95% confidence limits) for the best parsimonious predictive models for 20‐year incidence of coronary heart disease and peripheral arterial disease (sexes combined). CHD indicates coronary heart disease; hs, high‐sensitivity; NT‐pro‐BNP, N‐terminal prohormone of brain natriuretic peptide; PAD, peripheral arterial disease; ***R***‐, reciprocal of … ; SIMD, Scottish Index of Multiple Deprivation score (socioeconomic status); 25OHD, 25‐hydroxyvitamin D.

**Table 5:** Ranking of Hazard Ratios (shown with 95% Confidence Limits) in Descending Order (Binary Then Continuous) for the Best and Extended ASSIGN Models, for Coronary Heart Disease and Peripheral Arterial Disease

	Coronary Heart Disease: Best Model			
	Men 1847	Women 1251	All 3098	Rank
Binary				
Diabetes mellitus^a^	2.15 (1.63; 2.83)	2.19 (1.59; 3.02)	2.21 (1.79; 2.71)	1
Sex: male/female^a^			1.99 (1.72; 2.13)	2
Family history of CHD^a^	1.35 (1.22; 1.49)	1.52 (1.36; 1.70)	1.42 (1.32; 1.53)	3
Continuous				
Age^a^	1.34 (1.26; 1.42)	1.48 (1.37; 1.60)	1.38 (1.32; 1.44)	1
Total cholesterol^a^	1.37 (1.28; 1.46)	1.23 (1.14; 1.32)	1.31 (1.25; 1.38)	2
hsTroponin I	1.19 (1.03; 1.35)	1.23 (1.12; 1.34)	1.21 (1.11; 1.31)	3
NT‐pro‐BNP	1.22 (1.15; 1.29)	1.21 (1.11; 1.31)	1.21 (1.15; 1.27)	3
Cotinine	1.20 (1.11; 1.30)	1.16 (1.06; 1.28)	1.19 (1.12; 1.26)	5
R*‐*Apolipoprotein‐A	1.15 (1.09; 1.22)	1.18 (1.10; 1.25)	1.16 (1.11; 1.22)	6
Waist circumference	1.17 (1.05; 1.30)	1.11 (1.02; 1.21)	1.15 (1.06; 1.25)	7
R‐height	1.12 (1.04; 1.19)	1.16 (1.06; 1.27)	1.14 (1.08; 1.19)	8
hsC‐reactive protein	1.08 (1.00; 1.17)	1.19 (1.09; 1.31)	1.13 (1.06; 1.19)	9
Systolic blood pressure^a^	1.13 (1.08; 1.19)	1.09 (1.03; 1.16)	1.12 (1.08; 1.16)	10
SIMD score (SES)^a^	1.07 (1.01; 1.12)	1.11 (1.05; 1.18)	1.09 (1.05; 1.13)	11
R‐years of education	1.08 (1.02; 1.14)	1.09 (1.01; 1.18)	1.08 (1.03; 1.12)	12
Cigarettes/day^a^	1.06 (0.98; 1.13)	1.13 (1.02; 1.24)	1.07 (1.02; 1.14)	13
R‐LDL‐cholesterol	1.08 (1.01; 1.15)	1.05 (0.97; 1.14)	1.06 (1.01; 1.12)	14
Cystatin‐C	1.07 (1.02; 1.13)	1.04 (0.98; 1.10)	1.06 (1.02; 1.09)	14
Homocysteine	1.08 (1.02; 1.13)	1.02 (0.94; 1.11)	1.06 (1.02; 1.10)	14
Insulin	1.04 (1.00; 1.09)	1.07 (1.02; 1.12)	1.05 (1.02; 1.09)	17

	Peripheral Arterial Disease: Best Model			
	Men 291	Women 208	All 499	Rank
Binary				
Diabetes mellitus^a^	3.56 (2.14; 5.92)	3.49 (1.80; 6.76)	3.38 (2.23; 5.13)	1
Tobacco smoker	1.74 (1.18; 2.58)	2.76 (1.68; 4.53)	2.15 (1.58; 2.93)	2
Sex: male/female^a^			1.59 (1.30; 1.96)	3
Family history of CHD^a^	1.51 (1.18; 1.94)	1.35 (1.02; 1.79)	1.44 (1.20; 1.74)	4
Continuous				
Age^a^	1.55 (1.34; 1.80)	1.65 (1.38; 1.97)	1.62 (1.45; 1.81)	1
hsC‐reactive protein	1.37 (1.19; 1.58)	1.51 (1.27; 1.80)	1.42 (1.27; 1.59)	2
Systolic blood pressure^a^	1.30 (1.15; 1.45)	1.28 (1.13; 1.45)	1.30 (1.19; 1.42)	3
Expired carbon monoxide	1.20 (1.05; 1.37)	1.32 (1.13; 1.54)	1.24 (1.13; 1.38)	4
Cotinine	1.26 (1.04; 1.53)	1.22 (0.96; 1.55)	1.24 (1.06; 1.45)	4
SIMD score (SES)^a^	1.26 (1.12; 1.42)	1.20 (1.04; 1.38)	1.24 (1.13; 1.36)	6
Lipoprotein (a)	1.12 (0.94; 1.34)	1.56 (0.96; 2.53)	1.21 (1.04; 1.42)	7
R‐25OHD (adj)	1.32 (1.10; 1.59)	1.05 (0.81; 1.37)	1.20 (1.03; 1.41)	8
NT‐pro‐BNP	1.24 (1.08; 1.42)	1.16 (0.95; 1.42)	1.19 (1.06; 1.34)	9
Glucose	1.22 (1.12; 1.33)	1.12 (1.01; 1.23)	1.18 (1.11; 1.24)	10
Triglycerides	1.03 (0.91; 1.17)	1.34 (1.13; 1.58)	1.16 (1.04; 1.29)	11
Cystatin‐C	1.10 (0.99; 1.22)	1.10 (0.97; 1.25)	1.10 (1.02; 1.19)	12

	Coronary Heart Disease: Extended ASSIGN			
	Men 1847	Women 1251	All 3098	Rank
Binary				
Diabetes mellitus^a^	2.19 (1.66; 2.89)	2.20 (1.59; 3.04)	2.24 (1.82; 2.76)	1
Sex: male/female^a^			2.04 (1.82; 2.27)	2
Family history of CHD^a^	1.36 (1.23; 1.50)	1.53 (1.36; 1.71)	1.43 (1.32; 1.54)	3
Continuous				
Age^a^	1.34 (1.27; 1.42)	1.48 (1.37; 1.60)	1.38 (1.32; 1.45)	1
Total cholesterol^a^	1.39 (1.31; 1.49)	1.23 (1.14; 1.32)	1.33 (1.26; 1.39)	2
hsTroponin I	1.21 (1.04; 1.38))	1.24 (1.12; 1.36)	1.22 (1.13; 1.33)	3
NT‐pro‐BNP	1.21 (1.14; 1.28)	1.20 (1.10; 1.30)	1.20 (1.14; 1.26)	4
Cotinine	1.17 (1.09; 1.26)	1.14 (1.04; 1.25)	1.16 (1.09; 1.23)	5
C‐reactive protein	1.11 (1.03; 1.21)	1.23 (1.13; 1.34)	1.16 (1.09; 1.24)	5
Systolic blood pressure^a^	1.15 (1.09; 1.21)	1.11 (1.05; 1.17)	1.14 (1.09; 1.18)	7
R‐Apolipoprotein‐A	1.11 (1.04; 1.20)	1.16 (1.05; 1.28)	1.14 (1.08; 1.20)	7
SIMD score (SES)^a^	1.08 (1.03; 1.14)	1.12 (1.06; 1.19)	1.10 (1.06; 1.15)	9
R‐Height	1.08 (1.01; 1.15)	1.14 (1.04; 1.25)	1.10 (1.04; 1.16)	9
R‐years of education	1.09 (1.02; 1.15)	1.09 (1.01; 1.18)	1.09 (1.03; 1.14)	11
Cigarettes/day^a^	1.06 (0.99; 1.14)	1.13 (1.02; 1.25)	1.08 (1.02; 1.14)	12
Insulin (non‐fasting)	1.06 (1.02; 1.10)	1.08 (1.03; 1.13)	1.07 (1.04; 1.10)	13
Cystatin‐C	1.08 (1.03; 1.14)	1.04 (0.99; 1.10)	1.07 (1.03; 1.10)	14
R‐LDL‐cholesterol	1.08 (1.01; 1.14)	1.04 (0.97; 1.12)	1.06 (1.01; 1.12)	15
R‐HDL cholesterol^a^	1.06 (1.00; 1.14)	1.03 (0.94; 1.12)	1.05 (1.00; 1.10)	16

	Peripheral Arterial Disease: Extended ASSIGN			
	Men 291	Women 208	All 499	Rank
Binary				
Diabetes mellitus^a^	3.58 (2.15; 5.96)	3.23 (1.68; 6.23)	3.36 (2.21; 5.10)	1
Sex: male/female^a^			1.79 (1.43; 2.22)	2
Family history of CHD^a^	1.50 (1.17; 1.92)	1.36 (1.03; 1.81)	1.44 (1.20; 1.73)	3
Continuous				
Age^a^	1.56 (1.34; 1.82)	1.67 (1.38; 2.01)	1.62 (1.44; 1.81)	1
Thiocyanate	1.42 (1.13; 1.80)	1.36 (1.03; 1.80)	1.43 (1.20; 1.71)	2
hsC‐reactive protein	1.37 (1.18; 1.59)	1.49 (1.25; 1.77)	1.41 (1.26; 1.59)	3
Systolic blood pressure^a^	1.34 (1.17; 1.55)	1.36 (1.18; 1.58)	1.36 (1.23; 1.51)	4
SIMD score (SES)^a^	1.31 (1.16; 1.47)	1.21 (1.05; 1.40)	1.28 (1.17; 1.41)	5
Lipoprotein (a)	1.12 (0.94; 1.34)	1.58 (0.94; 2.63)	1.21 (1.04; 1.41)	6
Expired carbon monoxide	1.14 (0.99; 1.32)	1.29 (1.10; 1.52)	1.20 (1.08; 1.33)	7
Cotinine	1.19 (0.95; 1.51)	1.25 (0.96; 1.63)	1.20 (1.00; 1.44)	7
NT‐pro‐BNP	1.24 (1.08; 1.42)	1.14 (0.92; 1.42)	1.19 (1.05; 1.35)	9
R‐25OHD (raw)	1.27 (1.06; 1.52)	1.09 (0.80; 1.47)	1.19 (1.02; 1.41)	9
Glucose	1.23 (1.12; 1.34)	1.13 (1.02; 1.24)	1.18 (1.12; 1.25)	11
Triglycerides	1.04 (0.90; 1.19)	1.31 (1.09; 1.59)	1.15 (1.02; 1.29)	12
R‐DBP	1.09 (0.94; 1.27)	1.12 (0.97; 1.32)	1.11(1.00; 1.23)	13
Cystatin‐C	1.10 (0.99; 1.22)	1.10 (0.97; 1.25)	1.10 (1.02; 1.19)	14
Cigarettes/day^a^	1.04 (0.88; 1.21)	1.11 (0.89; 1.39)	1.06 (0.93; 1.20)	15
Total cholesterol^a^	1.06 (0.91; 1.23)	1.02 (0.85; 1.21)	1.05 (0.94; 1.17)	16
HDL cholesterol^a^	1.05 (0.94; 1.18)	0.94 (0.80; 1.11)	1.01 (0.91; 1.11)	17

This table replicates data for the best parsimonious models of CHD and PAD in the Figure for the sexes combined, but additionally gives sex‐specific data for these models, and everything is then repeated for the extended ASSIGN parsimonious models. Hazard ratios are per standard deviation change for continuous variables, some log transformed (see Table 1).

a ASSIGN risk score factors.

### C‐Statistic and Net Reclassification Improvement

Finally the c‐statistic and net reclassification improvement (NRI) were calculated to see what difference was made in specific prediction of CHD and PAD, using the new models as against the historical ASSIGN cardiovascular risk factors (see Table 6). The original 9 ASSIGN risk factors gave high c‐statistics in both CHD and PAD, paradoxically higher in PAD, although it was a minority contributor in numbers of end points to the original derivation of the ASSIGN score.^15^ The new models showed improved c‐statistics and substantial NRIs for both end points, considerably more so for PAD. Differences between the “best” and the “extended ASSIGN” models were modest and even inconsistent.

**Table 6:** C‐Statistic and Net Reclassification Improvement (with 95% Confidence Limits) From Old to New Predictive Models

	Coronary Heart Disease			Peripheral Arterial Disease		
	Men	Women	Total	Men	Women	Total
	1847	1251	3098	291	208	499
C‐statistic						
ASSIGN variable model	0.690	0.734	0.723 (0.713; 0.734)	0.798	0.837	0.821 (0.795; 0.847)
Best model	0.712	0.750	0.741 (0.730; 0.751)	0.836	0.875	0.854 (0.828; 0.880)
Extended ASSIGN	0.712	0.750	0.740 (0.730; 0.751)	0.838	0.875	0.855 (0.829; 0.881)
Change in c‐statistic from ASSIGN variable to improved models						
Best model	0.022	0.016	0.018 (0.010; 0.025)	0.038	0.037	0.034 (0.023; 0.044)
Extended ASSIGN	0.021	0.016	0.017 (0.013; 0.021)	0.040	0.037	0.035 (0.024; 0.046)
Net reclassification improvement from ASSIGN variable to improved models						
Best model	0.069	0.072	0.056 (0.032; 0.080)	0.115	0.363	0.231 (0.148; 0.313)
Extended ASSIGN	0.064	0.077	0.048 (0.025; 0.071)	0.127	0.372	0.186 (0.107; 0.265)

The final pattern of risk factors in the “best” models differs from the original ASSIGN panel in both CHD and PAD, with the recruitment of new factors and the downgrading of some classic factors, and there are noteworthy differences between all CHD and all PAD cases in these analyses, despite the 57% overlap of cases during follow‐up.

## Discussion

### PAD, the Cardiovascular Iceberg

PAD is prevalent worldwide, causing serious morbidity and contributing to the burden for health services associated with the pandemics of cigarette smoking and diabetes mellitus, with amputation a dreaded complication.^1^, ^2^, ^3^, ^4^, ^5^ It trails behind CHD and stroke, the lion and tiger of CVD, as an identifiable killer; like an iceberg, it mostly lacks visibility.

PAD causes arterial narrowing and a diminished ankle‐to‐brachial blood pressure index, (ABI). So PAD is now defined as an ABI of ≤0.9,^5^, ^23^ which facilitates international research with increasing prevalence and incidence compared to clinical diagnosis.^1^ In SHHEC clinical incidence was 6 times greater for CHD than PAD, but questionnaire positive prevalence results at baseline were 2.6 times higher. ABI measurements were not used, so their effect on comparative prevalence and incidence is unknown.

The epidemiology of CHD is dominated by cohort studies, following healthy participants for disease; PAD is dominated by case‐control studies from vascular clinics, follow‐up of diabetic populations, or screening studies involving ABI, sometimes repeated.^6^, ^7^ ABI suffers the problems of other screening tools.^24^, ^25^ In simultaneous assessment of disease and risk factors, there is potential confusion of cause and effect.^26^ Some large‐cohort studies of PAD had a limited panel of risk factors at recruitment and concluded that risk factors for CHD and PAD are the same.^1^, ^27^

### Merits and Limitations of the Study

The advantages include these:

1. Cohort design, with random population sampling across Scotland, covering classic and novel risk factors.
2. Twenty‐year follow‐up by national record linkage of multiple‐cause death certification and hospital diagnoses during an era of high incidence of CVD.
3. Measurement of additional biomarkers on all participants after the end, using archived serum.
4. Prospective design minimizes both observer bias and confusion of cause and effect.

The disadvantages are these:

1. Predictive factors were measured only once in each participant.
2. Although 90.1% were linked to national disease records in follow‐up, unlinked participants could be persistently too healthy to do so, or unhealthy failures.
3. The latest follow‐up of end points was to the end of 2009, but completion of laboratory work took until 2014, so there was a gap, but 20 years was achieved.
4. Diagnoses of CHD and PAD were based on episodes, numbering thousands, throughout Scotland, so independent validation was impracticable.
5. Onset of CHD or PAD need not be followed by death or hospital admission, or, if they were, probabilities of their being recorded differ.
6. Deep‐frozen serum, collected nonfasting, valid for many assays, was inappropriate for others where fasting specimens or plasma are preferable. For particular assays frozen serum might deteriorate over 14 to 20 years.
7. Other risk factors might have been measured but were not or were measured in only a minority of cases in SHHEC.
8. Some factors were recorded in SHHEC but not analyzed in addition to the 45 listed (for example, food frequency, alcohol, exercise, fatty acids).
9. Finally, the nature of multiple imputation is multiple iteration.^19^ Repeating the procedure with different iterations produces different answers through the play of chance. Contrast the “best” and “extended ASSIGN” models in the diseases (see Table 5).

### Methodological Issues: HRs by Sex and in PAD Versus CHD

HRs by sex for the same disease end point appear very similar; most differences are small (see Table 4). Female HRs are generally higher than male. The largest differences in HR occur when both are high. When the 2 HRs are either side of 1.0, 1 or both tend to lack statistical significance. Largest differences by sex in HR tend to diminish when simply age adjusted (Table 4) or when additionally adjusted for ASSIGN variables (Table 4), and even further in the parsimonious models after adjustment for other factors in the model (Table 5). There appears not to be an overall pattern except for the higher female than male values. These can be explained by a similar mechanism to HRs for PAD being greater than for CHD (next paragraph). The observed sex differences do not test a previous, or suggest a new, hypothesis, so they are reported without any testing of their differences statistically. Such tests are reserved for the overall disease differences with the sexes combined.

Table 3 shows numerous examples of variables where the HR for PAD is significantly higher than that for CHD. These disease differences apply also to parsimonious modeling (although HRs for individual factors are not strictly comparable after mutual adjustment). Factors with lower HRs and wider confidence limits crossing 1.0 lack statistical significance, more likely in PAD with fewer end points. In the Figure the lowest significant HR is 1.10 for PAD but 1.05 for CHD. But this is only a partial explanation.

HRs are ratios. The HR for tobacco smoking (Table 3) is 5.12 (4.14, 6.33) for PAD versus 1.70 (1.58, 1.83) for CHD and 6.76 (4.88, 9.35) versus 2.63 (2.16; 3.21) for diabetes mellitus. Because the incidence of clinical PAD overall is one sixth that for CHD, the risk of PAD in nonsmoking nondiabetic participants is necessarily small. Of nonsmoking nondiabetics at recruitment, 15.9% in 20 years developed CHD versus only 1.1% PAD. Of smoking diabetics, 43.3% developed CHD versus 17.3% PAD: CHD risk increased 2.7‐fold, versus PAD risk 16‐fold. This explains higher c‐statistics and net reclassification improvements in PAD than CHD (see Table 6).

### Methodological Issues: Why Factors Disappear, Reverse Their HR Gradients, or Substitute for Each Other

Adjustment, after age and sex, for other ASSIGN factors reduces some HRs to insignificance, even reversal. Adjustment in modeling also leads to some gradient reversals. This applies to closely correlated factors—the stronger survive, causing rivals to diminish or turn tail—but chance will operate between those closely matched (see Table 7 for correlation coefficients of competing variables). For example:

1. In the best model for CHD, total cholesterol has a more positive HR than in Table 3, whereas that for LDL‐cholesterol is now reversed.
2. In the above model, HDL‐cholesterol, associated with a number of factors, is relegated to insignificance; a major detractor presumably is apolipoprotein‐A.
3. Both total cholesterol and HDL‐cholesterol are lost from the PAD best model where other lipids have taken over.
4. Nonfasting glucose contributes to PAD, whereas nonfasting insulin contributes to CHD.
5. Adjusted 25OHD survives into the best model for PAD; *raw* values into extended ASSIGN.
6. Fibrinogen disappears from all models in the presence of hsCRP.
7. Cigarette equivalent dose, an ASSIGN factor, appears in all models except the best model for PAD, where it is ousted by the yes/no tobacco smoker question, which in turn is supplanted in extended ASSIGN for PAD by cigarette dose, plus all 3 smoking biochemistry factors, with thiocyanate now dominant.

These interactions explain why factors that appear significantly predictive in our Table 3 and in many publications do not appear in the parsimonious models in the Figure and Table 5—they have not been so challenged. Maybe factors we have not measured will someday challenge those that we have.

### Symptom Questionnaires and Baseline ECG as Predictors

Data on sensitivity and specificity of the symptom questionnaires and ECG are shown in Table 2. A minority of clinical events over 20 years were anticipated by these items. More surprising is the low percentage of baseline questionnaire positives who subsequently developed clinical disease (the positive predictive value). Either the questionnaires are nonspecific or preclinical atheromatous disease may improve or fail to progress. Earlier cross‐sectional studies gave the Edinburgh claudication questionnaire high sensitivity and specificity.^12^

### Specific Predictors and the Overall Picture of Contrasting Hierarchies

Linked to the question of which factors are in or out of the contrasting models for CHD and PAD, is the ranking of those factors within each model, even those present in both (see Figure, Table 5). Although diabetes mellitus, sex, family history of CHD, and age lead the hierarchy for both end points, with cotinine nearing the top and cystatin‐c nearing the bottom, there is incongruity in other leaders. In PAD, total cholesterol and hsTroponin I, found in CHD, are supplanted by hsCRP and systolic blood pressure, suggesting that inflammation outranks lipids and is a real advance predictor and not secondary to severe arterial damage.^26^

#### Diabetes Mellitus and Insulin Resistance

Diabetes mellitus was the dominant factor in all models in terms of HR. However, the percentage of diabetics in the baseline population was unusually low (1.5%), contributing a small percentage of PAD cases during follow‐up–40/499 (8.0%). Even with incident diabetes mellitus codiagnosed with PAD cases by the end of follow‐up, it was 137/499 (27.5%). Results for CHD were 102/3098 (3.3%) and 512/3098 (16.5%). So diabetes mellitus was important, but not the dominant cause of PAD, as is sometimes believed. It may become so as its prevalence increases. We did not have fasting specimens or measure HbA_1c_,^28^, ^29^ but our results for nonfasting insulin and glucose suggest an additional role for insulin resistance.

#### Tobacco Smoking

All 5 smoking variables are interrelated and predictive of both CHD and PAD (Tables 3, 4, and 7). Two smoking variables, cigarette dose and cotinine, suffice for the CHD models, whereas the best PAD model includes 3, and extended ASSIGN for PAD includes 4 (Table 5). Although interrelated, they independently contributed to enhanced prediction in the presence of others. Cotinine appeared the most powerful smoking biomarker for CHD, and thiocyanate for PAD (Table 3).

**Table 7:** Pearson Correlation Coefficients Between Associated/Competing Variables in the SHHEC Population, Sexes Combined

Lipids	HDL‐C	Non‐HDL	Triglyceridess	LDL‐C	Apo‐A	Apo‐B	Lipo (a)
Total cholesterol	0.191	0.940	0.320	0.566	0.167	0.792	0.183
HDL‐cholesterol		−0.156	−0.409	0.067	0.787	−0.208	0.039
Non‐HDL‐cholesterol			0.462	0.558	−0.099	0.872	0.176
Triglycerides				−0.004	−0.194	0.438	−0.014
LDL‐cholesterol					−0.044	0.445	0.045
Apolipoprotein A						−0.055	0.045
Apolipoprotein B							0.160
Smoking variables	ExpCO	Thiocyanate	Cotinine				
Cigarettes per d	0.736	0.764	0.817				
Expired carbon monoxide		0.747	0.765				
Thiocyanate			0.795				
Inflammatory	Fibrinogen						
hsC‐reactive protein	0.413						
Vitamin D	Raw^a^						
25OHD adjusted^a^	0.865						
Renal related	Creatinine						
Cystatin‐C	0.457						

All results are highly statistically significant. Apo‐A indicates apolipoprotein A; Apo‐B, apolipoprotein B; HDL, high‐density lipoprotein; hs, high sensitivity; LDL, low‐ density lipoprotein; SHHEC, Scottish Heart Health Extended Cohort; 25OHD, 25‐hydroxyvitamin D.

a 25‐Hydroxyvitamin D before and after adjusting for seasonal variation using month of measurement.

Tobacco smoking was a dominant risk factor for PAD. Although 45.7% of all SHHEC participants were tobacco smokers at recruitment, it was 55.0% for future CHD, and 77.8% for future PAD. Childhood smoking which is not analyzed here, has been reported elsewhere as an independent predictor for PAD.^30^

#### Physical Measurements

BMI was omitted from the ASSIGN cardiovascular score^15^ as weak and inconsistent between the sexes. Physical measurements were reexamined with this longer follow‐up of SHHEC. Examined alone (Tables 3 and 4) BMI is predictive in both sexes for CHD but not for PAD. Other physical measurements are also inconsistent between the end points. Waist circumference survives into 1 CHD model but not both, whereas height enters both. Systolic blood pressure is predictive in all 4 models and is the only physical factor in the PAD best model; its stronger weighting in the extended ASSIGN model for PAD is counterbalanced by a negative gradient for diastolic blood pressure, as also seen in Table 3 after adjustment for ASSIGN variables but not before. This suggests a role for pulse pressure, which fell out of this model but is reported elsewhere,^31^ and is evidence of aging and loss of elasticity in the arterial tree.^32^ Others have found BMI predictive for PAD, but with smokers and nonsmokers separated.^33^ In SHHEC smokers of both sexes had BMI averaging 1.0 kg/m^2^ less than in nonsmokers, possibly confounding our finding that BMI was not predictive.

#### Lipids

Addition of variables to the earlier ASSIGN 9 changes significant lipids for both end points. In CHD, for both models, total cholesterol remains significant, with apolipoprotein A an interloper along with LDL‐cholesterol (weak but reversing its gradient from Table 3), but HDL‐cholesterol is squeezed into insignificance. In the PAD models, both total cholesterol and HDL‐cholesterol lack significance although both are predictive in isolation (Table 3); but lipoprotein (a) becomes a contributory lipid^34^, ^35^ along with nonfasting triglycerides. Disappearance of HDL‐cholesterol was discussed earlier. The dominance of total cholesterol in the CHD models and its disappearance in PAD suggest differences in pathogenesis. A recent histological analysis of occlusive PAD lesions in amputation specimens reports the absence of lipids, but these were end‐stage and presumably followed medication.^36^ PAD is common in heterozygous familial hypercholesterolemia, which is against serum cholesterol having no role at all.^37^

#### Newer Biomarkers

hsTroponin I, NT‐pro‐BNP, and hsCRP (in that order) enter the parsimonious models for CHD. For PAD, hsCRP outranks NT‐pro‐BNP, but hsTroponin I fails to qualify. In both, cystatin‐C figures low down in the list, knocking out a weaker creatinine. Using the same database as here, we recently concluded that 25OHD, although predictive in isolation, failed to be independently predictive for CVD after multiple adjustment.^38^ Negative results for CHD and positive results for PAD are intriguing in these analyses, with adjusted 25OHD weakly predictive in the best model but raw 25OHD supplanting it in extended ASSIGN. Previously published results are commonly cross‐sectional, a problem for ascertaining causality.^39^, ^40^

### Clinical and Etiological Significance of These Results

The results of this study are of interest in causation but do not necessarily mean that separate risk scores for different cardiovascular end points would be useful. Clinically diagnosed PAD is delayed, less frequent (3.2% over 20 years in those disease‐free at measurement), and often follows the onset of CHD, so its independent prediction is of lesser clinical interest than CVD as a whole. Risk factors that we measured are not all routinely available and would involve costs and delays if made so. Omission of any 1 would alter the models, necessitating recalculation. Some factors are positive predictors in isolation but negative in combination with others, creating possible confusion. Clinicians most concerned with risk of PAD are diabetes mellitus physicians; diabetics under care are the subject of other cohort studies. For generalists assessing the apparently healthy, our results suggest that although diabetes mellitus is a major risk factor, it is not the most prevalent. Tobacco smoking is, with self‐report being reinforced by biochemical measures of intake and of inflammation.

Prediction of CHD and PAD are improved by adding new factors. Not surprisingly in CHD, for which 8 of the 9 ASSIGN factors remain in the new models, the c‐statistic for the ASSIGN 9 alone is high at 0.723 and modestly improved in the 2 parsimonious models, For PAD this is 7 out of 9; the c‐statistic is very high for the ASSIGN model (0.821), and there is a large change in the 2 new models (0.854 and 0.855) with associated NRI results.

These results place newer biomarkers into context (as well as some now‐neglected older ones) but demonstrate once again the importance of diabetes mellitus and smoking in the etiology of PAD, in contrast to CHD, where blame is more evenly distributed. Family history of CHD and socioeconomic status, adopted into the ASSIGN cardiovascular risk score in 2007,^15^ maintain their importance as predictors for both end points, against potential interlopers.

The question of why atheromatous disease in vessel walls of different arteries at different sites, exposed to the same blood medium, should have disparate determinants, is unanswered, but there must be differences in the reactivity of these arterial walls.^6^, ^7^, ^25^ These results take the question a stage further: is atherothrombosis 1 disease or a cluster of overlapping diseases, with cholesterol in CHD largely supplanted by inflammation,^26^, ^28^, ^41^ diabetes mellitus, and smoking in PAD?

## Sources of Funding

The Scottish Heart Health Extended Cohort (SHHEC) was funded by the Scottish Health Department Chief Scientist Organization, the British Heart Foundation, and the FP Fleming Trust. The MORGAM collaboration was funded by the European Commission Seventh Framework Programme, references FP7/2007‐2013 (HEALTH‐F4‐2007‐2014113, ENGAGE; HEALTH‐F3‐2010‐242244, CHANCES). The MORGAM Biomarker Study (serum biomarkers in the MORGAM populations) was funded by the Medical Research Council, London (reference G0601463, No 80983). The BiomarCaRE Project (Biomarkers for Cardiovascular Risk Assessment in Europe) was funded by the European Commission Seventh Framework Programme FP7/2007‐2013 (reference HEALTH‐F2‐2011‐278913). Funding bodies had no role in the planning of the study, analyses, interpretation, writing or publication of the manuscript, or recruitment of participants.

## Disclosures

None.
